# Acute Pulmonary Embolism Presenting With Angina and a Positive Cardiac Stress Test

**DOI:** 10.7759/cureus.11006

**Published:** 2020-10-17

**Authors:** Maham A Waheed, Mazin Khalid, Arsalan Talib Hashmi, Yury Malyshev, Sergey Ayzenberg

**Affiliations:** 1 Internal Medicine, Miamonides Medical Center, Brooklyn, USA; 2 Cardiology, Maimonides Medical Center, Brooklyn, USA

**Keywords:** pulmonary embolism, thrombectomy, chest pain

## Abstract

Acute pulmonary embolism (PE) is a commonly missed clinical entity. Prompt diagnosis of PE and the initiation of anticoagulation therapy is vital for the reduction of patient mortality. Recognizing initial electrocardiogram manifestations can aid rapid diagnosis and prompt management. The most common EKG findings associated with PE are sinus tachycardia, S1Q3T3 pattern, presence of T wave inversions in V1-V3 associated with the presence of right ventricular (RV) dysfunction, and right bundle branch block. These findings, while specific, are modestly sensitive and not always present. The gold standard of diagnosis is computerized tomographic angiography and ventilation and perfusion (V/Q). Here we present a patient who presented with symptoms mimicking angina with EKG changes in his stress test, prompting coronary angiography, which showed obstructive coronary artery disease requiring revascularization. Subsequently, further evaluation revealed a saddle pulmonary embolism that necessitated pulmonary thrombectomy.

## Introduction

Acute pulmonary embolism (PE) is a commonly missed clinical entity and is often discovered after rapid patient demise. A retrospective study of 188 patients found that 12% of patients have pulmonary emboli on autopsy findings, out of which only 9% of cases had been diagnosed before demise [1]. Prompt diagnosis of PE and the initiation of anticoagulation therapy are vital for reducing patient mortality [2]. Although ventilation and perfusion (V/Q) scans and computerized tomographic angiography (CTA) are confirmatory in establishing a diagnosis of PE, recognizing initial electrocardiogram (EKG) manifestations can aid rapid diagnosis and prompt management [3]. The most common EKG findings associated with PE are sinus tachycardia, S1Q3T3 pattern, presence of T wave inversions in V1-V3 associated with the presence of right ventricular (RV) dysfunction, and right bundle branch block. These findings, while specific, are modestly sensitive and not always present [4]. Here we present a patient with acute PE who presented with symptoms mimicking angina with EKG changes in his stress test, prompting coronary angiography, which showed obstructive coronary artery disease requiring revascularization. Subsequently, further evaluation revealed a saddle pulmonary embolism that necessitated pulmonary thrombectomy.

## Case presentation

A 78 year-old-man with a past medical history of essential hypertension (HTN) presented to his primary care physician with chest pain for one week, retrosternal, radiating to the left arm, dull in nature, brought by walking half a block and alleviated by rest. The patient denied any associating symptoms. He provided us with a treadmill nuclear stress test report that revealed downsloping ST-segment depressions in leads I, II, V4-6, and had to stop after five minutes and 24 seconds due to chest pain and dyspnea. The nuclear imaging did not show perfusion defects. He was referred to our cardiology clinic for further management. He was observed to have profound shortness of breath and clenched his chest upon walking a few steps in our facility. Given his history and stress test result, he was rushed to the emergency department (ED) for urgent coronary angiography. 

**Figure 1:**
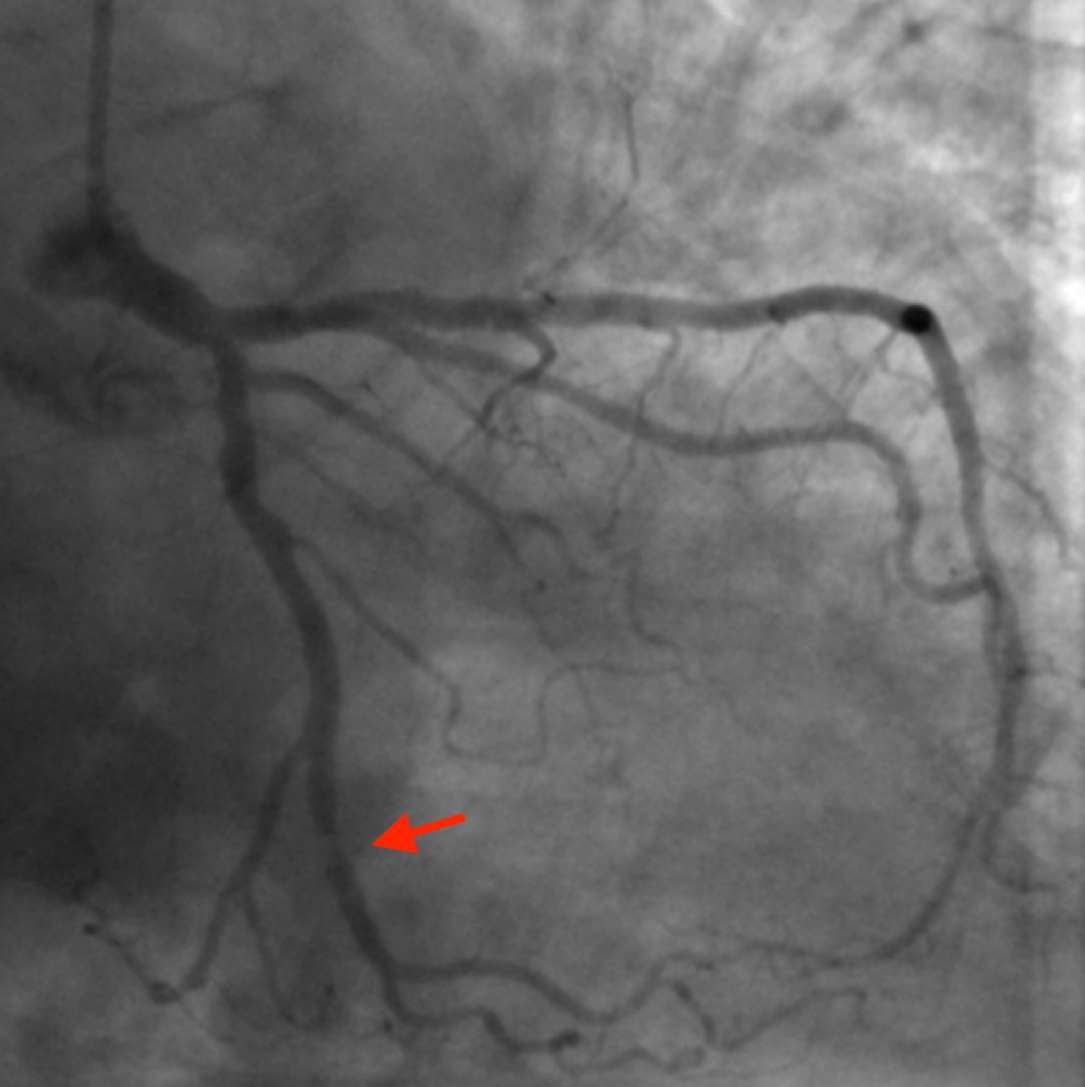
Coronary angiography image with the red arrow showing stenosis to the obtuse marginal artery Right anterior oblique caudal projection showing an area of severe stenosis of the obtuse marginal artery.

On arrival to the ED, he was afebrile, had heart rate (HR) 92 beats/min, respiratory rate (RR) 19/min, blood pressure (BP) 145/94, and was saturating 94% on room air. Serum troponin I was elevated to 0.04 ng/mL (normal value is 0.00 - 0.02 ng/ml). Urgent cardiac catheterization revealed 40% stenosis in the left main coronary, 50% stenosis in the proximal and distal right coronary artery. The third obtuse marginal artery had 99% stenosis, which was revascularized using a drug-eluting stent (Figure 1). His symptoms were out of proportion to the angiographic findings; hence we sent d-dimer, which was significantly elevated - 3257 (normal value 0 - 500). He remained dyspneic on ambulation; therefore, we obtained an urgent chest CTA, which revealed saddle pulmonary embolus extending into bilateral upper, bilateral lower, and right middle lobe pulmonary arteries and their branches, without infarct. Bilateral lower extremity venous duplex scan revealed acute deep vein thrombus (DVT) in the Left popliteal vein.

**Figure 2:**
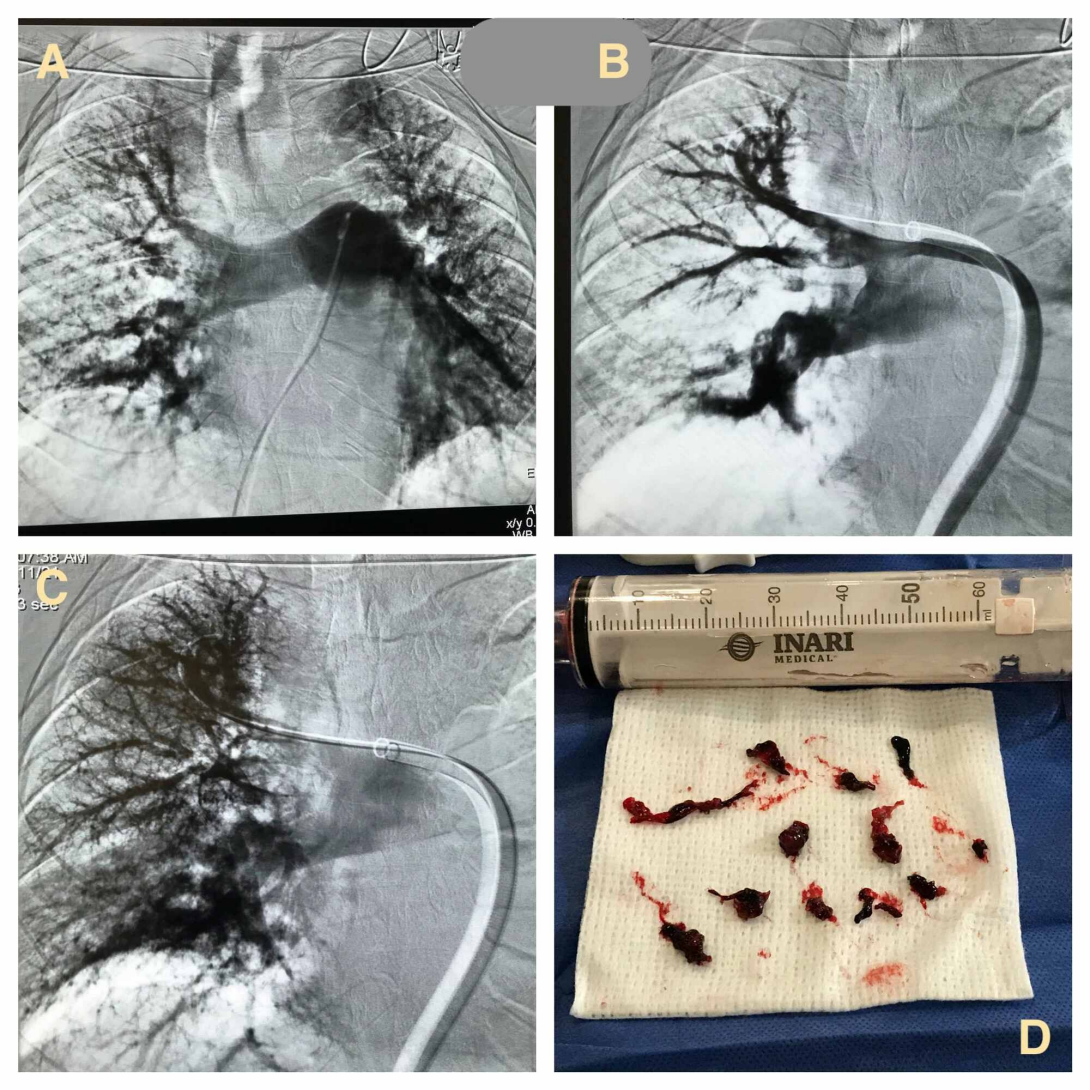
Pulmonary angiography A: bilateral pulmonary angiography showing filling defect more predominant on the right lung. B: right lung pulmonary angiography showing diffuse filling defects more pronounced in the middle lobe. C: right lung angiography after thrombectomy showing improvement of the perfusion. D: thrombectomy syringe with multiple clots extracted from the pulmonary arteries.

Furthermore, transthoracic echocardiogram (TTE) revealed normal left ventricular ejection fraction, increased pulmonary artery systolic pressure (46 mmHg), and moderately enlarged right ventricle with reduced systolic function. Despite optimal anticoagulation therapy initiation, the patient continued to exhibit shortness of breath on minimal exertion and had occasional de-saturations on room air; therefore, he underwent pulmonary angiography and catheter-directed thrombectomy. Pulmonary artery angiography revealed a moderate clot burden in the right pulmonary artery (Figure 2). 

The patient’s shortness of breath resolved post thrombectomy. He was discharged home on aspirin, clopidogrel, and oral apixaban therapy for anticoagulation. 

## Discussion

Saddle PE resulting from the embolization of large occlusive thrombus to the pulmonary artery is associated with high morbidity and mortality. More often than not, patients with PE present with symptoms that mimic acute coronary syndromes such as chest pain, dyspnea, syncope, and hemoptysis [5]. ST segment and T wave changes are the most common EKG abnormalities seen in acute PE. S1Q3T pattern is often considered the pathognomonic EKG abnormality for acute PE [6]. Electrocardiographic ST segment elevations and new T wave inversions are also commonly seen, especially in massive PE patients [7, 8]. Our patient had evidence of ST segment depressions on his nuclear stress test. His typical chest pain and the clinical picture were concerning stable angina secondary to underlying coronary artery disease. However, the lack of correlation between the angiographic findings and clinical presentation added to the persistence of exertional dyspnea and transient desaturations aroused PE's concern. It has been reported that patients with untreated PE have a 30% mortality rate, while those who receive treatment have a mortality rate of 2-8% [2]. Therefore, early recognition and treatment of PE are vital to mortality reduction in these patients. 

Traditionally, PE has been treated initially with injectable anticoagulation using intravenous unfractionated heparin or subcutaneous fondaparinux [9]. Recently direct oral anticoagulants, such as oral rivaroxaban, have shown similar efficacy and are accepted as initial therapy [10]. The use of apixaban as first-line therapy in patients with acute PE and DVT was shown to have the added benefit of reduced hospitalizations, as seen in the Apixaban for the Initial Management of Pulmonary Embolism and Deep‐Vein Thrombosis as First‐Line Therapy (AMPLIFY) trial [11]. Patients with saddle PE and evidence of hemodynamic compromise benefit from urgent thrombectomy.

## Conclusions

Acute saddle PE can present with angina symptoms and produce ST depressions on stress testing. Physicians should keep the diagnosis of PE in mind when addressing their patients. We should pay special attention to the clinical correlation between the angiographic findings and clinical manifestation as it can be a clue for the diagnosis of massive PE.
